# Initial viral cycle threshold values in patients with COVID-19 and their clinical significance

**DOI:** 10.1186/s40001-022-00729-5

**Published:** 2022-06-28

**Authors:** Salma AlBahrani, Mohammed Alghamdi, Nawaf Zakary, Arulanantham Zechariah Jebakumar, Samirah Jamaan AlZahrani, Mohamed Hany ElGezery, Khaled Omar Abdallah, Jaffar A. Al-Tawfiq

**Affiliations:** 1 King Fahad Military Medical Complex, Dhahran, Saudi Arabia; 2 Infectious Disease Unit, Specialty Internal Medicine, Dhahran, Saudi Arabia; 3 Vice Deanship of Postgraduate Studies and Research, Prince Sultan Military College of Health Sciences, Dhahran, Saudi Arabia; 4 Specialty Internal Medicine and Quality Department, Dhahran Health Center, Johns Hopkins Aramco Healthcare, Room D-0032, Building 61, P.O. Box 76, Dhahran, 31311 Saudi Arabia; 5 Infectious Disease Division, Department of Medicine, Indiana University School of Medicine, Indianapolis, IN USA; 6 Infectious Disease Division, Department of Medicine, Johns Hopkins University School of Medicine, Baltimore, MD USA

**Keywords:** SARS-CoV-2, Cycle threshold, Coronavirus disease 2019, COVID-19, Viral load

## Abstract

**Background:**

The connection between initial viral cycle threshold (Ct) values of the SARS-CoV-2 with symptoms and hospital course is not clearly studied.

**Methods:**

This is a retrospective study of hospitalized COVID-19 patients from Jun 1st 2020 to March 30th, 2021 examining the relationship between initial viral cycle threshold (Ct) values of SARS-CoV-2 as obtained from nasopharyngeal samples. The clinical presentations and outcomes were analyzed in relation to the initial Ct values.

**Results:**

The study included 202 hospitalized COVID-19 patients with a mean age (± SD) of 54.75 (± 15.93) and 123 (60.9%) males and 79 (39.1%) females. Of all the patients, the most frequent comorbidity was diabetes mellitus (95; 47%) and the most frequent symptoms were fever (148; 73.3%) and cough (141; 69.8%). There was no significant difference in relation to underlying conditions, clinical presentation, radiographic and laboratory data among those with low, medium and high Ct values. The mean Ct values showed no statistical change over the 10-month study period.

**Conclusions:**

Initial SARS-CoV-2 Ct values did not show any association with clinical symptoms and did not predict the need for mechanical intubation or death.

## Introduction

The severe acute respiratory syndrome coronavirus 2 (SARS-CoV-2) had resulted in a global pandemic with increasing number of cases and associated death [1]. The Kingdom of Saudi Arabia had also been involved with pandemic as early as March 2020 with the implementation of multiple steps to combat the pandemic [2–4]. There are many studies showing the risk of increased disease severity such as: age, body mass index (BMI), underlying medical diseases, clinical signs and symptoms, and laboratory data [5, 6]. In addition to comparisons between different pandemic waves in the country [7], there is an interest to investigate the association of initial and subsequent SARS-CoV-2 viral quantity as extrapolated from viral cycle threshold (Ct) values with clinical signs and symptoms as well as the need for hospital admission and possible death or recovery. A previous study showed no difference in the viral load kinetics among patients with different severity of COVID-19 [8]. One study of 5000 patients showed no statistical difference in viral loads between patients with or without symptoms [9]. A small study of 76 patients revealed that severe COVID-19 cases were associated with increased viral loads and duration of viral shedding in comparison to those with milder symptoms [10]. We are not aware of any studies examining the association between viral Ct values and clinical symptoms or outcome in Saudi Arabia. Thus, the current study investigates the relations between viral Ct values in patients with SARS-CoV-2 infection in association with different clinical parameters.

## Materials and methods

We conducted a retrospective study examining the relationship of initial viral Ct values and clinical symptoms and severity of COVID-19 and the correlation with viral Ct values over time. The severity of the disease was based on (1) respiratory rate > 30 breaths/min; (2) O2 saturation < 93% on room air; (3) a ratio of PaO2 to FiO2 of < 300 mm Hg and cases requiring intensive care were those who had (1) respiratory failure requiring mechanical ventilation; (2) hemodynamic shock; (3) multi-organ failure [11, 12]. We included hospitalized COVID-19 patients > 13 years of age between Jun 2020 and March 2021. We collected and analyzed age, gender, different symptoms, management, clinical course, laboratory data, and outcome (admission to the intensive care unit (ICU), survival or death). We compared the Ct values of the SARS-CoV-2 with these parameters.

The SARS-CoV-2 was detected using real-time reverse transcriptase polymerase chain reaction (RT-PCR). Abbott system (extraction and amplification/detection) or Roche instruments were used for extraction and amplification by Real Star SARS-CoV-2 RT-PCR kit Altona-Diagnostics). Both systems are based on full sample preparation (nucleic acid extraction and purification). The RT-PCR amplified two regions: RdRp and N-genes, or E and S genes, in the Abbott and Roche (Altons) systems, respectively. For the result of Roche samples, we used the S gene value as the E-gene considered as screening. We separated the Ct values for the SARS-CoV-2-specific target (ORFlab) into terciles based on the quantitative values. We then designated high viral load samples as the lowest Ct tercile, medium viral load samples as the middle tercile, and low viral load samples as the highest tercile. The Ct values were classified as: low Ct values < 25 (*n* = 40 Roche, *n* = 101 Abbott), medium with Ct values 25–30 (*n* = 39 Roche, *n* = 19 Abbott), and high Ct values of > 30 (*n* = 9 Roche, *n* = 0 Abbott) [13]. Roche and Abbott systems were in use in the hospital beginning of Jun 2020, but by the end of October 2020, Roche machine was not used for technical issues and the laboratory continued to use the Abbott system.

The study was approved by the IRB of the King Fahad Military Medical Complex (AFHER-IRB-2020–033).

### Statistical analysis

Statistical analyses were done using Windows Excel and the Statistical Package for Social Sciences (SPSS) 25 packages. The comparison between qualitative variables was dependent on the Chi-square and the quantitative variables by analysis of variance (ANOVA). We compared the different Ct values in relation to the demographics, clinical presentation and outcome. A significant *p* value was considered if < 0.05.

## Results

From Jun 1st, 2020 to March 30th, 2021, a total of 202 patients with COVID-19 were admitted with a mean age (± SD) of 54.75 (± 15.93) years, and 123 (60.9%) were males. The most frequent comorbidity was diabetes mellitus (95; 47%) and the most frequent symptoms were fever (148; 73.3%) and cough (141; 69.8%) (Table 1). Admission to the ICU was required for 84 (33.6%) of the patients.

**Table 1:** Underlying comorbidities and clinical presentations of included patients

Characteristics	Frequency	Percentage
Diabetes mellitus	95	47.0
Cardiac disease	43	21.3
Lung disease	23	11.4
COPD	3	1.5
Heart failure	11	5.4
ESRD	13	6.4
Hemodialysis	8	4.0
Cancer	3	1.5
Fever	148	73.3
Shivering	16	7.9
Shortness of breath	88	43.6
Chest pain	20	9.9
Wheezes	6	3.0
Cough	141	69.8
Hemoptysis	5	2.5
Sore throat	29	14.4
Headache	36	17.8
Myalgia	65	32.2
Vomiting	29	14.4
Diarrhea	34	16.8
Tachypnea	8	4.0
Respiratory distress	26	12.9
Oxygen saturation	100	49.5
Admitted to ICU	70	34.7
Single lobar infiltrate	20	15.0
Multi-lobar infiltrate	125	71.4

COPD: chronic obstructive pulmonary disease; ESRD, end-stage renal disease; ICU: intensive care unit

Of all the cases, 88 (43.5%) were tested with the Roche machines and the mean (± SD) of the viral Ct value was 23.95 ± 5.89. And 102 (59.4%) were tested with Abbott machine and had mean Ct value of 16.2 (± 7.74).

A comparison of the underlying comorbidities showed no significant difference between the three viral load (Ct values) groups in the two rt-PCR machines (Tables 2 and 3), apart from age where those with medium level of viral Ct values were younger in the Abbott group (Table 2).

**Table 2:** Comparison of baseline characteristics among the different Ct values in those who were tested using Abbott rt-PCR machine

Baseline characteristics	Low Ct value (*N* = 101)	Medium Ct value (*N* = 19)	*P* value
Age (mean, SD) years	57.05 (15.09)	50.79 (13.00)	0.04*
Male	63 (62.4)	11 (57.9)	0.71
Lung disease	8 (7.9)	3 (15.8)	0.28
Cardiac disease	22 (21.8)	4 (21.1)	0.94
Diabetes mellitus	53 (52.5)	7 (36.8)	0.21
COPD	2 (2.0)	0	0.54
Hemodialysis	4 (4.0)	2 (10.5)	0.23
ESRD	7 (6.9)	4 (21.1)	0.05
Heart failure	7 (6.9)	2 (10.5)	0.59
Contact with another person	33 (32.7)	5 (26.3)	0.59

COPD: chronic obstructive pulmonary disease; ESRD: end-stage renal disease

**Table 3:** Comparison of baseline characteristics among the different Ct values in those who were tested using Roche rt-PCR machine

Baseline characteristics	Low Ct value (*N* = 39)	Medium Ct value (*N* = 35)	High Ct value (*N* = 8)	*P*-value
Age in years	53.38 (18.28)	55.14 (16.07)	40 (12.56)	0.08
Male	20 (51.3)	25 (71.4)	4 (50.0)	0.18
Lung disease	6 (15.4)	6 (17.1)	0	0.46
Cardiac disease	6 (15.4)	10 (28.6)	1 (12.5)	0.31
Diabetes mellitus	15 (38.5)	17 (48.6)	3 (37.5)	0.65
COPD	1 (2.6)	0	0	0.57
Cancer	2 (5.1)	1 (2.9)	0	0.74
Hemodialysis	0	2 (5.7)	0	0.25
ESRD	0	2 (5.7)	0	0.25
Heart failure	0	2 (5)	0	0.25
Contact with another person	22 (56.4)	18 (51.4)	4 (50.0)	0.89

COPD: chronic obstructive pulmonary disease; ESRD: end-stage renal disease

**Fig. 1:**
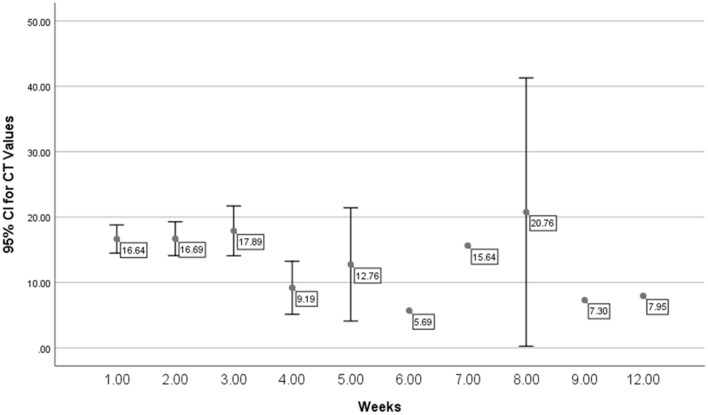
The mean and 95% CI of viral cycle threshold per study week with the 95% confidence intervals for patients tested using the Abbot rt-PCR machine

**Fig. 2:**
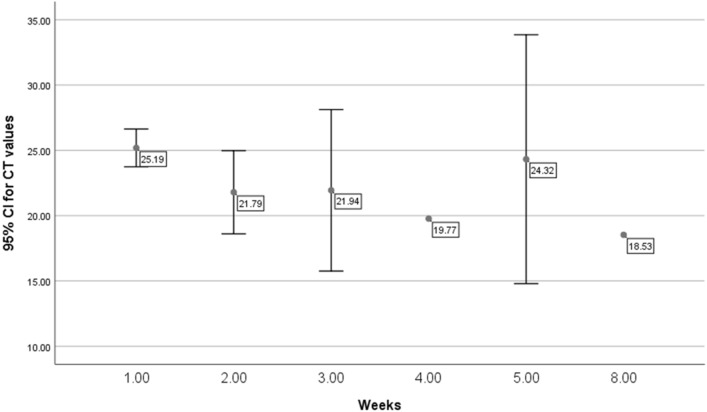
The mean and 95% CI of viral cycle threshold per study week with 95% confidence intervals for patients tested using Roche rt-PCR machine

Presenting symptoms were similar between the different viral Ct values (Tables 4 and 5). However, there was a statistical difference in the percentage with oxygen saturation < 93% in those with low viral Ct (57.4%) and those with medium viral Ct vale (31.6%) (P = 0.04) in the Abbott tested group but not in the Roche tested group (Tables 6 and 7). The laboratory findings and outcome were similar between the different Ct value groups (Tables 8 and 9). However, CRP was higher among those with medium viral Ct values than the other two groups in the Roche group (Table 9). Over the study period of 10 months, there was no statistically significant change in the mean Ct values per week (Figs. 1 and 2).

**Table 4:** Comparison of clinical symptoms among patients with different Ct values in those who were tested using Abbott rt-PCR machine

Symptoms	Low Ct value (*N* = 101)		Medium Ct value (*N* = 19)
Fever	74 (73.3)	12 (63.2)	0.37
Shivering	11 (10.9)	2 (10.5)	0.96
Shortness of breath	44 (43.6)	6 (31.6)	0.33
Chest pain	10 (9.9)	3 (15.8)	0.45
Wheezes	3 (3)	0	0.45
Cough	69 (68.3)	11 (57.9)	0.38
Hemoptysis	2 (2)	1 (5.3)	0.4
Sore throat	13 (12.9)	2 (10.5)	0.78
Headache	17 (16.8)	3 (15.8)	0.91
Myalgia	35 (34.7)	4 (21.1)	0.25
Vomiting	16 (15.8)	0	0.06
Diarrhea	15 (14.9)	2 (10.5)	0.62

**Table 5:** Comparison of clinical symptoms among patients with different Ct values in those who were tested using Roche rt-PCR machine

Symptoms	Low Ct value (*N* = 39)	Medium Ct value (*N* = 35)	High Ct value (*N* = 8)	P-value
Fever	30 (75)	30 (76.9)	7 (77.8)	0.97
Shivering	2 (5)	1 (2.6)	0	0.7
Shortness of breath	14 (35)	22 (56.4)	5 (55.6)	0.14
Chest pain	3 (7.5)	4 (10.3)	0	0.59
Wheezes	1 (2.5)	2 (5.1)	0	0.68
Cough	32 (80)	25 (64.1)	7 (77.8)	0.27
Hemoptysis	0	2 (5.1)	0	0.28
Sore throat	7 (17.5)	7 (17.9)	1 (11.1)	0.88
Headache	7 (17.5)	7 (17.9)	2 (22.2)	0.95
Myalgia	11 (27.5)	15 (38.5)	2 (22.2)	0.47
Vomiting	6 (15)	6 (15.4)	1 (11.1)	0.95
Diarrhea	6 (15)	10 (25.6)	1 (11.1)	0.39

**Table 6:** Comparison of clinical variables among patients with different Ct values in those who were tested using Abbott rt-PCR machine

Signs	Low Ct value (*N* = 101)		Medium Ct value (*N* = 19)
Tachypnea	5 (5)	1 (5.3)	0.95
Respiratory distress	13 (12.9)	2 (10.5)	0.78
Single lobar infiltrate	11 (15.3)	0	0.15
Multi-lobar infiltrate	63 (68.5)	10 (62.5)	0.64
Oxygen saturation < 93%	58 (57.4)	6 (31.6)	0.04*
Admitted to ICU	40 (39.6)	9 (47.4)	0.53

**Table 7:** Comparison of clinical variables among patients with different Ct values in those who were tested using Roche rt-PCR machine

Variables	Low Ct value (*N* = 39)	Medium Ct Value (*N* = 35)	High Ct value (*N* = 8)	*P*-value
Tachypnea	1 (2.5)	1 (2.6)	0	0.89
Respiratory distress	4 (10)	6 (15.4)	3 (33.3)	0.2
Oxygen saturation	17 (42.5)	19 (48.7)	5 (55.6)	0.73
Admitted to ICU	9 (22.5)	13 (33.3)	4 (44.4)	0.34
Single lobar infiltrate	5 (19.2)	4 (16.7)	0	0.87
Multi-lobar infiltrate	24 (70.6)	27 (84.4)	6 (100)	0.16

**Table 8:** Comparison of laboratory data among patients with different Ct values in those who were tested using the Abbott rt-PCR machine

	Abbot	*N*	Mean	Std. deviation	95% CI for Mean		*P*-value
					Lower	Upper	
WBC	Low Ct value	99	6.15	3.27	5.31	7.17	0.06
	Medium Ct value	18	7.77	4.02	5.26	10.03	
PMN	Low Ct value	100	4.44	3.01	3.64	5.24	0.22
	Medium Ct value	19	5.38	3.36	3.49	7.12	
Lymph%	Low Ct value	100	20.97	12.92	16.68	23.93	0.59
	Medium Ct value	19	19.28	8.87	15.39	26.04	
Lymph	Low Ct value	99	1.47	2.74	0.99	1.38	0.98
	Medium Ct value	18	1.49	0.75	0.93	2.03	
Plat	Low Ct value	100	228.37	129.15	185.64	261.57	0.71
	Medium Ct value	19	240.16	113.25	168.35	339.47	
ALT	Low Ct value	65	47.58	40.88	37.56	61.52	0.13
	Medium Ct value	14	88.66	203.61	− 54.77	255.1	
AST	Low Ct value	95	45.56	33.71	35.24	54.35	0.047
	Medium Ct value	18	86.61	187.18	− 50.58	271.51	
LDH	Low Ct value	98	321.60	138.82	281.44	360.75	0.55
	Medium Ct value	16	345.77	214.54	179.03	473.33	
D-dimer	Low Ct value	96	1.55	2.18	0.94	2.36	0.014*
	Medium Ct value	17	3.58	6.13	0.53	5.81	
Ferritin	Low Ct value	96	633.65	773.43	352.7	630.71	0.88
	Medium Ct value	17	664.88	756.33	123.41	1216.28	
CRP	Low Ct value	97	79.28	69.37	58.39	96.72	0.26
	Medium Ct value	16	101.21	83.14	38.13	155.49	
Procalcitonin	Low Ct value	96	0.79	2.96	− 0.06	2.05	0.51
	Medium Ct value	17	0.31	0.35	0.15	0.68

**Table 9:** Comparison of laboratory data among patients with different Ct values in those who were tested using the Roche rt-PCR machine

Variable	Ct value	*N*	Mean	Std. deviation	95% Confidence interval for mean		*P*-value
					Lower bound	Upper bound	
WBC range	Low	40	5.23	2.50	4.43	6.03	0.13
	Medium	38	6.72	3.88	5.44	7.99	
	High	9	5.79	2.67	3.74	7.84	
PMN range	Low	40	3.47	2.01	2.83	4.12	0.03*
	Medium	39	4.69	3.21	3.65	5.73	
	High	9	8.42	13.45	− 1.91	18.76	
Lymph% range	Low	40	23.00	14.15	18.48	27.53	0.93
	Medium	38	21.97	12.73	17.79	26.16	
	High	9	21.62	11.65	12.66	30.58	
Lymph range	Low	40	1.20	0.67	0.99	1.42	0.72
	Medium	39	1.36	1.09	1.01	1.71	
	High	9	1.28	0.64	0.79	1.77	
Plat range	Low	40	215.69	86.29	188.10	243.29	0.61
	Medium	39	236.83	98.59	204.87	268.79	
	High	9	227.76	106.47	145.91	309.60	
ALT range	Low	17	46.36	30.79	30.53	62.19	0.18
	Medium	8	54.10	39.43	21.13	87.07	
	High	3	89.33	56.05	− 49.90	228.56	
AST range	Low	40	50.17	34.22	39.22	61.11	0.89
	Medium	39	52.70	63.22	32.21	73.19	
	High	8	59.30	43.10	23.27	95.33	
LDH range	Low	39	304.99	148.97	256.70	353.28	0.08
	Medium	39	385.71	171.43	330.14	441.28	
	High	8	357.95	114.86	261.92	453.97	
D-dimer	Low	39	1.18	1.69	0.63	1.73	0.2
	Medium	39	4.37	11.66	0.59	8.15	
	High	9	1.59	2.43	− 0.28	3.47	
Ferritin	Low	39	476.51	642.60	268.20	684.82	0.16
	Medium	39	923.46	1337.36	489.94	1356.98	
	High	8	720.24	651.14	175.87	1264.60	
CRP	Low	39	60.81	63.56	40.21	81.41	0.04*
	Medium	37	99.25	79.10	72.87	125.62	
	High	8	53.08	62.04	1.21	104.94	
Procalcitonin	Low	38	1.25	6.88	− 1.01	3.51	0.63
	Medium	37	3.61	17.56	− 2.25	9.46	
	High	9	0.14	0.12	0.05	0.23

## Discussion

In this study, we showed no difference in underlying characteristics or symptoms among the different Ct values of SARS-CoV-2 admitted patients, apart from few characteristics. Two studies of SARS-CoV-2 viral load showed correlation with disease severity [10, 14]. However, SARS-CoV-2 Ct values are not normally reported to the treating team. One study suggested that reporting Ct values may help identifying patients needing anti-viral therapy such as remdesivir [15]. The results from this study did not support the value of baseline viral load (Ct values) relative to disease severity. However, one study showed statistical association between Ct values of SARS-CoV-2 and initial symptoms, clinical spectrum, mortality and sequelae [16]. The current understanding of COVID-19 indicates that age and underlying medical conditions are indicative of poor outcome [17, 18]. In addition to these associations, few studies had also found association between Ct values and laboratory values including biomarkers in patients with COVID-19 infection [16, 19, 20]. In one prospective study, the calculated viral loads were independently associated with death [19] as well as predictor of death among patients who have or do not have malignancy [21]. The predictions of Ct values of the individual outcomes and prognosis are not clearly known. It would be interesting to add the Ct values to other predictors of mortality to examine whether such combination would be of additional prognostic value.

It had speculated that the occurrence of death later in the course of the disease might indicate that severe disease might not correlate with higher viral loads [22]. Previous studies indicated that the presence of SARS-CoV-2 in cultures correlates with Ct values < 24 [23] or < 33 [24]. In addition, viral loads had been correlated with severe disease and the risk of infectivity [13, 23]. There was a relationship between viral loads and increased mortality and the need for mechanical ventilation among high viral load (Ct < 25) patients (35% and 29%) compared to lower risks among low viral load (Ct > 30) with risks of 6% and 15% for death and mechanical ventilation, respectively [13]. Similar to our study, previous studies did not find an association of SARS-CoV-2 Ct values with disease severity [6, 25]. Other investigators find no correlation between viral Ct values and the presence of symptoms [26] and between inpatients and outpatients [27]. The difference between these studies might be related to the technique of sample collection, variation in the testing methods, variations in techniques and runs, and timing of the samples collected as reported previously [28]. In addition, timing of the testing and calculation of the Ct values in relation to symptoms would affect the level of the Ct values [29].

In this study, we also looked at the mean Ct values among the study population overtime. We had not detected any statistically significant change in the mean Ct values per week over the study period. In an interesting study, it was suggested that the viral Ct values correlates with the course of the pandemic with higher Ct values when the pandemic was decreasing and lower Ct values when the pandemic was increasing. It was suggested that calculation of the Ct values predict the evolution of the pandemic [30]. Another study also showed a connection between the population Ct values overtime and the course of the pandemic [31]. Thus, it had been suggested that the use of population Ct values as a proxy of the growth rate of the pandemic and the transmission in any given population or community [32–34] with wide variation overtime and among the different population [35].

This study had few limitations in addition to being a retrospective in design. There were different staff who obtained samples for PCR testing and this may had resulted in differences in techniques; however, all of them were trained and deemed competent. In addition, the Ct values were based on PCR tests taken on admission, however patients may have been admitted at different days from onset of symptoms and thus both Ct values and outcome may be influenced by the day of admission in relation to the onset of symptoms. We had not done serial testing and the data for admission to the hospital and the ICU were not correlated with the Ct values.

In conclusion, this study did not find any association of the initial viral Ct values and clinical symptoms or outcome in admitted COVID-19 patients. Similarly, another study did not reveal any association between initial or nadir Ct values and survival rate or mild/moderate versus severe/critical illness [36]. On the other hand, another study showed correlation between lower Ct values and mortality [37]. Further studies are needed to try to elucidate the dynamics of the viral Ct values and the pathogenesis of the disease in order to understand the disease and outcome.

## Data Availability

Data are available upon request.
